# Impact of Smoking on Cervical Histopathological Changes in High-Risk HPV-Positive Women: A Matched Case–Control Study

**DOI:** 10.3390/medicina61020235

**Published:** 2025-01-28

**Authors:** İlkan Kayar, Goksu Goc, Ferhat Cetin, Özer Birge

**Affiliations:** 1 Department of Obstetrics and Gynecology, Osmaniye State Hospital, 80000 Osmaniye, Turkey; 2 Department of Obstetrics and Gynecology, American Hospital, 10000 Prishtine, Kosovo; goksu.goc@gmail.com; 3 Department of Obstetrics and Gynecology, Private Osmaniye Park Hospital, 80010 Osmaniye, Turkey; ferhat_cetin@hotmail.com; 4 Department of Gynecological Oncology, Niger Turkey Friendship Hospital, Niamey G3PR+2JJ, Niger; ozbirge@gmail.com

**Keywords:** high-risk HPV, cigarettes, quantity of cigarette packs, colposcopy, endocervical curettage, histopathology

## Abstract

*Background and Objectives*: The aims of this study were to assess the impact of smoking on cervical histopathology in women with high-risk HPV types 16 and 18 (the most common types) utilizing comprehensive clinical data and to conduct a risk analysis based on smoking pack-years. *Materials and Methods*: Between 2022 and 2024, 1048 high-risk HPV-positive women aged 25 to 65 years were categorized into two groups: smokers and non-smokers. Data acquired from a histopathological examination of samples collected during a colposcopic evaluation of these women were compared individually regarding clinical and demographic factors, specifically age, gravida, parity, and alcohol consumption. Subsequently, the impact of prolonged and excessive smoking on histopathological cellular changes was assessed in women with the same characteristics. A case–control study was performed on 312 smokers and 312 non-smokers following mutual matching. *Results*: The women were matched one-to-one regarding gravida, parity, and alcohol consumption. Subsequently, they were paired within a ±2-year age range. The mean age of the smoker group was 47.1 ± 8.8, while that of the non-smoker group was 47.2 ± 8.5 (*p*: 0.904). In all cases of high-risk HPV positivity, the rate of normal cervical cytological results was 14% in women who smoked and 29% in women who did not smoke. The LGSIL, HGSIL, ASC-H, and AGC-NOS rates were elevated in the smoker group, and a statistically significant difference was observed between the two groups in terms of abnormal cervical cytological results (*p* < 0.001). After a colposcopic biopsy, the smoker group exhibited higher rates of HGSILs, LGSILs, AGC-NOS, and CIS pathological lesions (28% vs. 23%), whereas the non-smoker group exhibited higher rates of chronic cervicitis (23% vs. 16%). However, no statistically significant difference was found between the two groups (*p*: 0.092). In a comparison of endocervical curettage (ECC) samples, it was observed that the HGSIL, CIS, and AGC-FN rates in the smoker group were almost the same as those in the non-smoker group. However, the LGSIL histopathology results (32% vs. 18%) were higher, and the rate of negativity with no pathology was higher in the non-smoker group (72% vs. 59%). A statistically significant difference in ECC histopathology was noted between the two groups (*p* < 0.001). An ROC analysis conducted between smoking pack-years and the colposcopic and endocervical curettage biopsy results revealed that the cutoff value for the colposcopic abnormal histopathological results increased, with 40% sensitivity and 76% specificity above 20 pack-years (AUC: 0.592 and *p*: 0.025). Additionally, the abnormal histopathology rates for endocervical curettage exhibited 81% sensitivity and 32% specificity above 13 pack-years (AUC: 0.586 and *p*: 0.008). The rate of abnormalities in the colposcopic biopsy results was 2.19 times higher for individuals with over 20 pack-years, and the rate of abnormalities in the ECC results was 2.08 times higher for those with over 13 pack-years; additionally, statistically significant results were obtained (*p*-values of 0.027 and 0.008, respectively). *Conclusions*: The most important cause of neoplastic changes in the cervix uteri is high-risk HPV infection, with evidence indicating that prolonged excessive smoking significantly exacerbates the persistence and progression of HPV infection, thereby influencing neoplastic changes in the cervix uteri. It is crucial for women to cease smoking in order to eradicate HPV infection from the body.

## 1. Introduction

Cervical cancer ranks as the fourth most prevalent cancer among women across all age demographics globally [1]. According to the GLOBOCAN global cancer statistics produced by the International Agency for Research on Cancer (IARC), in 2020, 604,127 women were diagnosed with cervical cancer, with 341,831 deaths attributable to the disease [2].

According to the GLOBOCAN statistics for Turkey, in 2020, cervical cancer ranked 19th among the 35 types of cancer in the country, with 2532 new diagnoses and 1245 related deaths [2].

Considering the conditions of Turkey in terms of cervical cancer, an achievable target is free community-based screening starting at age 30 and ending at age 65. The population to be screened is based on individuals registered with family medicine. It is repeated every five years with an HPV (human papillomavirus) or Pap smear test using an invitation method. Screening is terminated in women aged 65 or over whose last two HPV or Pap smear tests are negative. The HPV vaccine, which is the most effective method for primary protection against cervical cancer in Turkey, is not included in the routine free vaccination calendar. Upon the recommendation of relevant branch physicians, two doses are administered to girls aged 9–15, and three doses are administered to girls aged 15 or over [3].

Recently, it has been recommended to evaluate radical and minimally invasive surgical, medical, and advanced immunotherapy treatment methods for cervical cancer, which especially affects young women and continues to be significantly prevalent despite HPV vaccination campaigns all over the world [4].

The natural development of cervical carcinogenesis is believed to commence with the initial infection of the metaplastic epithelium in the cervical transformation zone by oncogenic HPV (human papillomavirus). Nonetheless, the majority of HPV infections disappear naturally within 2–4 years, with just a small percentage advancing to low- or high-grade cervical intraepithelial neoplasia (CIN) [1,5].

Consequently, with HPV infection, some additional factors, such as immune-suppressing infections, pharmacological treatments, chronic diseases, and smoking, are believed to facilitate the advancement of the neoplastic process in the cervix uteri epithelium [6].

The association between smoking and squamous intraepithelial neoplasia in the cervix uteri, as well as the potential progression to cervix uteri cancer with persistence, has been known for a long time. In 1977, Winkelstein first stated that smoking may be a risk factor for cervical cancer [7]. International epidemiological studies indicated that smoking correlates with an elevated risk of squamous cell carcinoma, particularly in the respiratory and genito-urinary systems [8]. Several cohort studies reported high rates of smoking ranging from 27.8% to 32.4% [9,10], and these publications suggest that smoking may be protective against HPV infection. However, another study stated that long-term or lifelong smoking increases the persistence of HPV infection [11].

Dysplastic changes in the transformation zone of the cervix uteri are primarily caused by HPV infection; nonetheless, it is noted that squamous intraepithelial neoplasia may persist and potentially advance to neoplastic transformation, particularly in individuals with a history of prolonged and active smoking, which is recognized as a contributing risk factor. Cigarette smoking has been identified as a cofactor of HPV in the etiology of cervical cancer [12]. The incidence of HPV correlates with active smoking, particularly the intensity of smoking [13]. Studies restricted to HPV-positive women have confirmed that the association between smoking and its effects is dose-dependent and ceases after the cessation of smoking [7].

Physiopathological studies have revealed the presence of harmful nicotine and its derivatives from smoking in the physiological mucoid material produced by cervix uteri cells [14]. The immunoactive Langerhans cells in these physiological protective secretions are reduced in number due to the toxic effects of smoking, leading to the development of immunosuppression against HPV infection. HPV infection is said to induce permanent and progressive carcinogenesis due to a diminished immune response, particularly in the cervix uteri [15,16].

Two studies identified a markedly elevated risk of high-grade squamous intraepithelial lesions (HSILs) and cervical cancer in HPV-positive women; however, Syrjänen et al. proposed that smoking constitutes an independent risk factor only for oncogenic HPV infection and not for high-grade CIN [8,17,18].

Most studies examining the correlation between smoking and cervical cancer have concentrated solely on active smoking. A pooled analysis of pairs from multicenter case–control studies conducted by the International Agency for Research on Cancer (IARC) indicated that passive smoking might not constitute an independent risk factor for invasive cervical cancer in the absence of active smoking [18]. The literature indicates that prolonged and continuous passive exposure to cigarette smoke elevates the risk of squamous intraepithelial neoplasia in the cervix uteri and cervical cancer [19,20].

A study assessing the correlation between passive smoke exposure, active smoking, and cervical intraepithelial neoplasia failed to establish a consistent relationship due to various limitations, including a small sample size of non-smokers, concurrent histories of active and passive exposure to cigarette smoke, and insufficient data regarding HPV and sexual behavior. Alongside smoking, aspects pertaining to sexual activity significantly contribute to the onset of this malignancy. It has been asserted that the early initiation of sexual activity, multiple sexual partners, and certain infections are significant contributors to the development of this cancer [21].

In our society, smoking prevalence is rising, particularly among women at a young age; thus, we sought to elucidate the isolated impact of extensive pack-year active smoking on the histopathological alterations observed in biopsies from the cervix uteri. This was achieved by correlating clinical and demographic risk factors in women with high-risk HPV-positive results during cervix uteri cancer screening as part of the national screening program and by referring to the existing literature.

## 2. Materials and Methods

This study included a total of 1048 women who sought treatment at our clinics from January 2022 to 2024 after routine cervical cytological screening (BD SurePath™ (Franklin Lakes, NJ, USA) Liquid-Based Cytology) or presenting gynecological problems and were identified as positive for high-risk HPV (using the digene^®^ Hybrid capture 2 HPV DNA Test (QIAGE, Germantown, MD, USA) (types 16 and 18)) during the examination. Following the acquisition of consent from our hospital’s ethical committee (date: 12 December 2024; number: E-774.99-262357757), data from the electronic archive system were retrospectively analyzed. Consent for participation in this study was acquired from all women in accordance with the Declaration of Helsinki.

Following clinical examinations and cytological, histological, and laboratory assessments of all patients, a total of 1048 women were included, comprising 411 smokers and 637 non-smokers, all diagnosed with squamous intraepithelial neoplasia in the cervix uteri. The pathology laboratory of the same centers conducted a histopathological examination of samples obtained via a colposcopic examination due to cervical squamous intraepithelial neoplasia. The histological investigation revealed that women with high-grade lesions underwent LEEP conization (loop electrosurgical excision procedure) and ECC (endocervical biopsy); the follow-up of the women with negative surgical margins is currently ongoing.

This study’s inclusion criteria were individuals aged 25 to 65; individuals eligible for routine cytological screening; active smokers or non-smokers; and individuals positive for HPV types 16 and 18.

This study’s exclusion criteria consisted of patients who were pregnant; those aged under 25 or over 65; individuals with primary or metastatic cancer in other organ systems; patients undergoing active treatment or follow-up; individuals with a history of colposcopic biopsy or conization; patients with a history of HIV (human deficiency virus), HSV (herpes simplex virus), CMV (cytomegalovirus), gonorrhea, chlamydia, or similar infectious diseases; individuals with a history of immunosuppression or organ transplantation; patients with active or past use of immunosuppressive medications; individuals with a history of lupus or other rheumatologic, hematologic, or autoimmune disorders; patients exposed to passive smoking; individuals with a history of previous chemotherapy or radiotherapy; and those unwilling to participate or whose clinical information was inaccessible.

A histopathological assessment was conducted on cervical biopsies obtained following a colposcopic examination in patients positive for HPV 16 and 18, as determined by cotesting and abnormal cervical cytology results in accordance with the Bethesda 2001 criteria after cervical cytological screening. All specimens dispatched to the pathology laboratory were assessed by the same histopathologist. The histopathological investigation revealed negative dysplasia, chronic cervicitis, AGC/NOS or FN (atypical glandular cells not otherwise specified or favor neoplasia), LGSIL/CIN1 (low-grade intraepithelial lesion/cervical intraepithelial neoplasia), LGSIL/CIN2, HGSIL/CIN2 (high-grade intraepithelial lesion/cervical intraepithelial neoplasia), HGSIL/CIN3, CIS (carcinoma in situ), and cervix cancer (classified as benign, malignant squamous intraepithelial neoplasia, or invasive).

A total of 1048 patients positive for HPV 16 and 18 were screened in this study; 411 were active smokers, and 637 were non-smokers.

The women were analyzed concerning variables such as age, menopausal status, body mass index (BMI), C-reactive protein (CRP), fasting blood glucose level, gravida, parity, history of abortion, educational attainment, contraceptive method, alcohol consumption, smoking habits, pack-year smoking duration, comorbid diseases, smear cytology, human papillomavirus (HPV) type, colposcopic assessment and biopsy findings, LEEP (loop electrosurgical excision procedure) conization, and endocervical biopsy results.

The selection of women to be included in the research from the control group was made in accordance with the determination of the sample size in the active smoker group. Initially, the characteristics of gravida, parity, and alcohol consumption were analyzed for similarity, and women exhibiting similarities were selected through one-to-one matching. Furthermore, ±2 matching was conducted based on age distribution. A total of 312 smokers and 312 non-smokers were selected, and a case–control study was conducted.

All women with high-risk HPV 16 and 18 positivity were compared in relation to similar data in terms of risk factors that may induce squamous intraepithelial neoplasia in the cervix uteri. The impact of smoking on the cervix uteri, particularly concerning high-grade cervical neoplastic changes, was assessed, with a comparative evaluation of this effect between the two groups based on the duration of active smoking and pack-year accumulation.

In this study, women with preinvasive and invasive carcinoma received surgical and additional treatments at our gynecological oncology center.

### Statistical Analyses

Statistical Methods: Data analysis was performed with IBM SPSS Statistics26. The normal distribution of continuous variables was checked using graphs (Q-Q plot) and normality tests (Kolmogorov–Smirnov), and it was found that they were not normally distributed. Therefore, the Mann–Whitney U test was used to compare independent groups. Descriptive statistics are presented as median and IQR (25–75) values. An ROC analysis was performed for continuous variables, and the most appropriate cutoff value was determined according to the Youden index. The distributions of categorical independent groups were compared using the Chi-Square Test, and odds ratios were calculated. Descriptive statistics are presented as numbers and percentages. In statistical comparison tests, a type 1 error margin was determined as α: 0.05, and two-tailed tests were applied.

## 3. Results

This study included 1048 women, comprising 411 smokers and 637 non-smokers, who were diagnosed with squamous intraepithelial neoplasia in the cervix uteri following a clinical examination and cytological, histopathological, and laboratory evaluations, as well as high-risk HPV (types 16 and 18).

The women were matched in terms of gravida, parity, and alcohol consumption. Subsequently, they were matched up to ±2 years in terms of age group. The mean age of the smoker group was 47.1 ± 8.8, while that of the non-smoker group was 47.2 ± 8.5 (*p*: 0.904). A total of 312 smokers and 312 non-smokers were included in this study (Table 1).

Upon an examination of the demographic, clinical, and cytopathological data of the women, it was observed that the body mass index (BMI) was elevated in the non-smoking group, revealing a statistically significant difference between the two groups (*p* < 0.001). No significant difference was detected between the two groups regarding fasting blood sugar, menopausal status, abortion history, dilatation curettage status, and contraceptive use status. The proportion of university graduates with higher education was statistically significantly higher among smokers than among non-smokers (12% vs. 3%) (*p* < 0.001). In the smoker group, 45% had comorbid diseases, while in the non-smoker group, the ratio was 27%, and a statistically significant difference was observed between the two groups (*p* < 0.001). The risk of comorbidity in the smoking group was 2.22 times higher than that in the non-smoking group (OR: 2.22, 95% CI: (1.58–3.1), and *p* < 0.001). In all cases with high-risk HPV positivity, the rates of normal cervical cytology were 14% in women who smoked and 29% in women who did not smoke. The LGSIL, HGSIL, ASC-H, and AGC-NOS rates were higher in the smoker group. A statistically significant difference was observed between the two groups regarding the rates of abnormal cervical cytology (*p* < 0.001) (Table 2).

A histopathological assessment was conducted on biopsies obtained following a colposcopic examination of high-risk HPV-positive women, and they were compared with regard to the cervical cytological findings. The rates of pathological lesions such as HGSILs, LGSILs, AGC-NOS, and CIS were higher in the smoker group (28% vs. 23%), but the rates of chronic cervicitis were higher in the non-smoker group (23% vs. 16%); nevertheless, no statistically significant difference was detected between the two groups (*p*: 0.092). When comparing endocervical curettage (ECC) samples, the HGSIL, CIS, and AGC-FN rates in the smoker group were nearly equivalent to those in the non-smoker group. However, LGSIL histopathology results were higher in the smoker group (32% vs. 18%), and the rate of negativity without pathology was also higher in the non-smoker group (72% vs. 59%). A statistically significant difference in ECC histopathology was observed between the two groups (*p* < 0.001). Upon comparing the risk ratios of smoking concerning the ECC histopathological results across the two groups—HGSILs, LGSILs, AGC-FN, CIS, and chronic cervicitis—it was found that the risk ratios for abnormal histopathological development were 1.79 times higher in the smoking group, and a statistically significant difference was observed between the two groups (OR: 1.79, 95% CI: (1.28–2.51), and *p*: 0.001). According to the colposcopic biopsy results and the loop electroexcisional procedure (LEEP) conization results, the rates of HGSILs, LGSILs, CIS, cervical cancer, and chronic cervicitis were 73% to 85% higher in the smoking group. Additionally, the CIS and cervical cancer rates were 3% and 1% higher than those in the non-smoking group, respectively; however, no statistically significant difference was noted between the two groups. In the histopathological assessment of ECC obtained following LEEP conization, the HGSIL rate was 38% in the smoking group and 11% in the non-smoker group, and a statistically significant difference was observed between the two groups in terms of abnormal histopathological lesions (*p*: <0.001) (Table 3).

**Figure 1:**
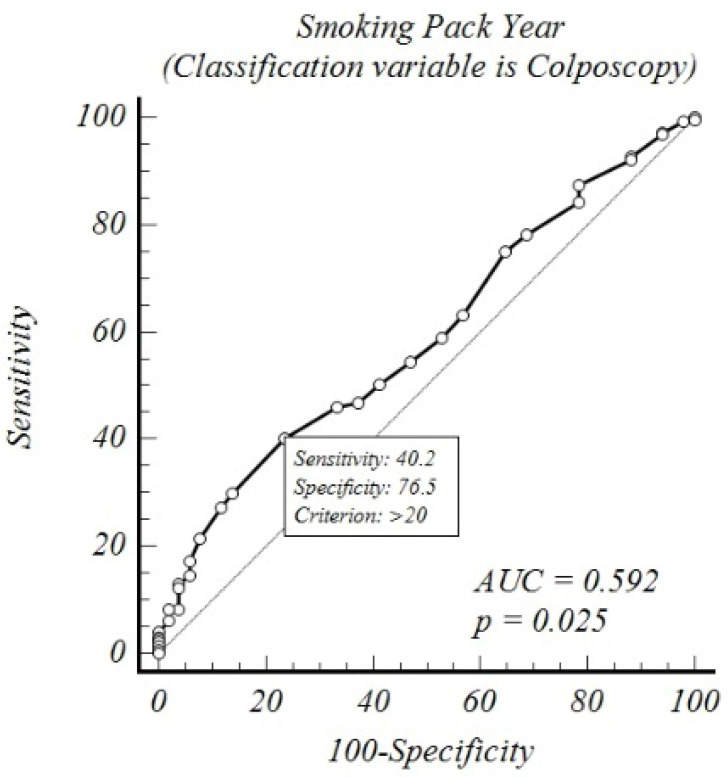
ROC analysis of colposcopic results according to smoking pack-years.

**Figure 2:**
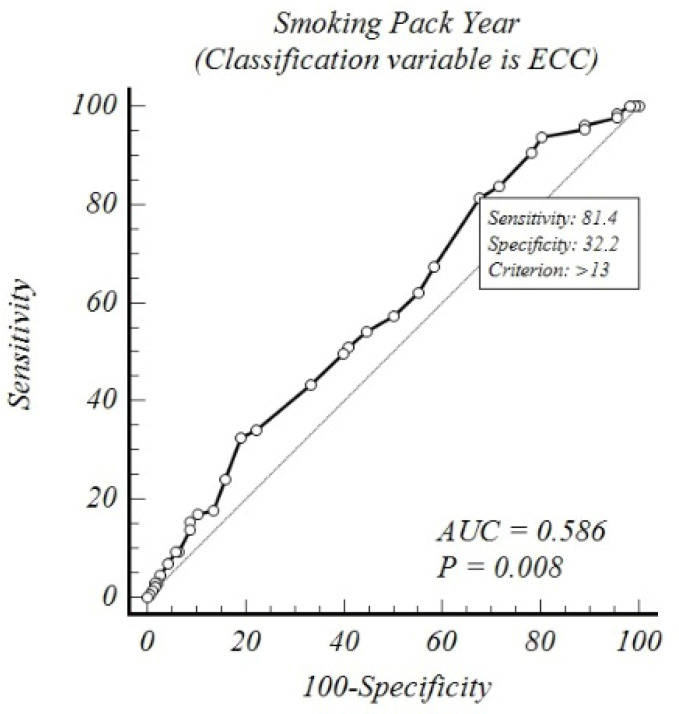
ROC analysis of endocervical curettage (ECC) results according to smoking pack-years.

An ROC analysis was conducted between smoking pack-years and the colposcopic and endocervical curettage biopsy results, revealing that the cutoff value for the colposcopic abnormal histopathological results increased to 40% sensitivity and 76% specificity above 20 pack-years (AUC: 0.592 and *p*: 0.025). In contrast, for endocervical curettage, abnormal histopathology rates increased to 81% sensitivity and 32% specificity above 13 pack-years (AUC: 0.586 and *p*: 0.008) (Table 4 and Figure 1 and Figure 2).

A risk analysis of the colposcopic biopsy results was conducted according to smoking pack-years, and abnormal histopathological results of HGSIL, LGSIL, AGC-NOS, CIS, and chronic cervicitis, which we accepted as normal, were considered as two groups. It was found that over 20 pack-years, the former group had a rate of 40%, higher than the rate of 24% for the latter group (*p*: 0.036), thus having a 2.19 times higher risk, and a statistically significant difference was detected between the two groups (OR: 2.19, 95% CI: (1.09–4.37), and *p*: 0.027) (Table 5).

A risk analysis of the endocervical curettage biopsy results was conducted according to smoking pack-years, and abnormal histopathological results of HGSILs, LGSILs, AGC/FN, CIS, and chronic cervicitis, which were considered normal, and negative results with no histopathological findings were considered as two groups. It was found that over 13 pack-years, the former group had a rate of 81%, higher than the rate of 68% for the latter group (*p*: 0.007); thus, there was a 2.08 times higher risk of developing abnormal histopathological lesions. Additionally, a statistically significant difference was detected between the two groups (OR: 2.08, 95% CI: (1.21–3.58), and *p*: 0.008) (Table 6).

## 4. Discussion

The overall findings of our study show that among individuals with high-risk HPV (types 16 and 18) positivity, the prevalence of normal cervical cytological results was 29% in non-smokers and 14% in smokers. Additionally, the LGSIL, HGSIL, ASC-H, and AGC-NOS rates were higher in the smoker group, and a statistically significant difference was observed between the two groups in terms of abnormal cervical cytological results (*p* < 0.001).

Pathological lesions, including HGSILs, LGSILs, AGC-NOS, and CIS, were detected at elevated rates following a colposcopic biopsy in the smoker group (28% vs. 23%), whereas chronic cervicitis rates were higher in the non-smoker group (23% vs. 16%). However, no statistically significant difference was noted between the two groups (*p*: 0.092). Upon a comparison of endocervical curettage (ECC) samples, it was observed that the HGSIL, CIS, and AGC-FN rates were nearly identical between the smoking and non-smoking groups. However, the LGSIL histopathology result was higher in the smoker group (32% vs. 18%), while the rate of negativity with no pathology was higher in the non-smoker group (72% vs. 59%). A statistically significant difference in ECC histopathology was noted between the two groups (*p* < 0.001).

The rate of abnormalities in the colposcopic biopsy results was 2.19 times higher for those with over 20 pack-years, and the rate of abnormalities in the ECC results was 2.08 times higher for those with over 13 pack-years; additionally, statistically significant results were obtained (*p*-values of 0.027 and 0.008, respectively).

We know that abnormal cytological and histopathological changes in the cervix uteri arise from high-risk human papillomavirus (hrHPV) infection. The majority of high-risk HPV infections are eradicated by the immune system; so, cervical intraepithelial neoplasia (CIN) does not appear to develop, and the underlying cause of CIN and subsequent invasive cervical malignancies in some high-risk HPV infections remains ambiguous. Among the contributing factors to this situation, active smoking or exposure to cigarette smoke is a significant behavioral factor, while immune system suppression, sexual behavior, other sexually transmitted infections, early age of coitus, multiple partners, and hormonal contraception represent additional risk factors [22].

Multiple explanatory hypotheses have been proposed concerning the association between smoking and cervical cancer, including a direct oncogenic impact on carcinogenesis from chemical toxic exposure or a carcinogenic effect resulting from the inhibition of cell-mediated immunity [23]. However, the precise role of smoking in cervical carcinogenesis remains unknown [24].

Despite many studies indicating a significant correlation between smoking, HPV infection, and CIN, the precise mechanism remains ambiguous. In studies that involve physiopathology, it has been asserted that nicotine derivatives may cause genomic damage to glandular mucoid cells in the cervix uteri through smoking-related toxins [14]. It has also been stated that exposure to cigarette smoke is associated with the activation of the p97 promoter in the cell proliferation cycle and the overexpression of E6/E7 oncogenes, particularly due to HPV viral infection, leading to the damage and excessive proliferation of epithelial cells, which results in an increase in neoplastic cells [25].

It has been stated that cigarette smoke and its nicotine metabolites inhibit innate and adaptive immune cells; reduce cytokine, natural killer cell, and immunoglobulin levels; create an immunosuppressive environment; and may increase susceptibility to infections, including persistent and progressive infections and associated neoplastic processes. It has also been noted that individuals with compromised antibody-mediated immunity who smoke have a heightened sensitivity to both acute and chronic infections, as well as an increased likelihood of persistent infections. HPV infection is reported to be more common among smokers and may persist and progress [26].

It has been shown that Langerhans cells, which are dendritic cells with immunological power, such as macrophages located in epithelial cells, decrease in number in smokers and cause local immunosuppression. The ligand that induces apoptosis associated with the tumor necrosis factor (TNF) of Langerhans cells also prevents HPV E6/E7 expression in cervix uteri epithelial cells through a cytotoxic effect. It has also been stated that HPV infection rapidly proliferates in smokers due to a reduction in the number of Langerhans cells, resulting in persistent infection and malignant dysplastic changes in epithelial cells [27]. Our study examined women with high-risk HPV (types 16 and 18) positivity who were divided into two groups, namely, smokers and non-smokers, regarding HPV infection, the predominant cause of cervical cytopathological changes. Consequently, we endeavored to elucidate the impact of smoking on HPV infection and, eventually, on the cervix uteri cells in isolation.

A total of 49 studies were included in a review conducted in 2021, addressing the following questions: “Is smoking an independent risk factor for the development of CIN (in different stages), and if so, what is the underlying cause of this risk?” and “Is smoking an independent risk factor for the development of cervical cancer, and if yes, what is the associated risk?” Attempts were made to answer these questions. Consequently, based on an analysis of all the studies reviewed, this systematic review and meta-analysis indicated that most studies demonstrated an elevated risk of cervical intraepithelial neoplasia (CIN) and cervical cancer among smokers. Previous studies investigated the correlation between smoking, CIN, and cervical cancer; however, the specific risk was not assessed, smoking and cigarette quantity were assessed subjectively, the different stages of CIN were not investigated, and CIN was not distinguished from cervical cancer. Upon reviewing all the studies, smoking was identified as a risk factor for the onset of cervical intraepithelial neoplasia (CIN) and cervical cancer; however, the exact contribution of smoking to cervical carcinogenesis remains unclear. It can be tentatively concluded that smoking increases the risk of histopathological abnormalities in the cervix uteri [28].

Despite the fact that there is a relationship between smoking and cancer, the pathophysiological relationship between smoking and HPV infection in the neoplastic process remains ambiguous. Numerous studies have indicated that the risk of HPV infection increases in women who smoke, including those who consume as few as 10 cigarettes daily [29,30]. Nevertheless, epidemiological studies rely on self-reported data, resulting in insufficient information regarding smoking habits, hence complicating the understanding of the true association between smoking and HPV [31,32].

In 2020, a prospective study found that smoking is a risk factor for cervical intraepithelial neoplasia lesions. However, there is limited information regarding the impact of smoking on the persistence of HPV infection, which is a transient infection, particularly in the young sexually active age group. Therefore, nicotine metabolites were analyzed in the urine of smokers, revealing that HPV-positive individuals with elevated levels of urine nicotine metabolites exhibited greater rates of p16/Ki-67 immunopositivity. Consequently, it was noted that CIN2/CIN3-positive high-grade cervical neoplastic lesions occurred at elevated rates due to increased p16/Ki-67 cellular biomarker tests in HPV-positive women with high levels of urinary nicotine metabolites linked to smoking, and HPV infection exhibited greater persistence, progression, and rapid proliferation. In smoking women, it has been asserted that markers disrupting the cell proliferation cycle, such as that of p16/Ki-67, must be significantly positive for HPV infection to induce neoplastic transformation and high-grade lesions, in contrast to non-smoking women [33].

Studies conducted on the cervix uteri and other body systems of smokers have measured nicotine and its metabolites, such as cotinine, as biomarkers in urine for objective assessments. The impact of nicotine metabolites on the persistence and progression of HPV infection in cancerous or precancerous lesions of the cervix uteri was used to validate these findings, particularly concerning the self-reported smoking behaviors of the participants. It has been observed that the rate of CIN3 and higher dysplastic lesions in the cervix uteri is statistically significantly elevated in individuals who smoke and exhibit nicotine metabolite levels exceeding 500 ng/mL, in comparison to those with levels below 500 ng/mL or non-smokers [31,32,34]. In our study, we could not quantify the level of nicotine metabolites in urine; however, after ascertaining the duration and quantity of cigarette consumption among our participants, we assessed their smoking history in pack-years and sought to elucidate the statistical impact of pack-years on high-grade cervical intraepithelial neoplastic lesions. While we endeavored to accurately disclose the number of pack-years, we acknowledge that self-reported smoking data should be corroborated with more objective biomarkers, long-term follow-ups, and studies involving a substantial sample size, akin to epidemiological studies. It is noted that high-grade lesions, particularly among smokers, escalate with the increase in the number of cigarette packs consumed.

The literature examining the relationship between smoking and HPV infection suggests that the healing rates of HPV infection are lower in smokers than in non-smokers and that dysplastic lesions exhibit rapid regression upon the cessation of smoking, resulting in a reduction in persistent HPV infection rates [35,36].

In another multicenter cross-sectional study where active and passive smoking was taken into account, a statistically significant relationship was observed between passive and active smoking and HPV infection and the risk of CIN 2 and cervical intraepithelial neoplasia (OR: 1.57 and 95% CI: 1.14–2.15 vs. OR: 1.99 and 95% CI: 1.02–3.88, respectively), and a less significant relationship was observed between passive smoking and general HPV infection (OR: 1.12 and 95% CI: 1.01–1.24). There was no statistically significant relationship between passive smoking and the frequencies of CIN2 and pathological lesions, irrespective of active smoking status (OR: 0.80 and 95% CI: 0.62–1.04). A statistically significant relationship was identified between active smoking and HPV infection, as well as between CIN2 and greater pathological changes in neoplastic cells among passive smokers (*p*: <0.001) [37]. Based on these findings, considering that high-risk HPV infection is a necessary precursor for cervical cancer, it was stated that high-risk HPV infection and CIN2+ have similar risk factors. In our study, all women were positive for high-risk HPV. We compared active smokers with non-smokers to determine whether an active smoking status and the quantity smoked, measured in pack-years, contribute to the development of high-grade cervical intraepithelial neoplastic lesions.

Our study revealed that the abnormal histopathology rates in the colposcopic biopsy results were elevated in the smoking group compared to the non-smoking group (28% vs. 23%). Furthermore, the abnormal histopathology rates in the ECC results were significantly higher in the smoking group (41% vs. 28%), demonstrating a statistically significant difference compared to the non-smoking group, which aligns with the literature (*p*: 0.001). Studies have indicated that both active and passive smoking serve as independent risk factors for cervical cancer. An analysis of case–control studies conducted by the International Agency for Research on Cancer (IARC) demonstrated that HPV infections in continuously active smokers can persistently progress, with a heightened risk of advancing to precancerous and invasive cancer, even in the absence of additional risk factors [38].

Multicenter and large cohort studies have indicated that smoking constitutes a significant risk factor for both CIN 3 and carcinoma in situ lesions, particularly in HPV serology positive women. The likelihood of developing CIN or SCC may be doubled due to the toxic metabolites present in cigarettes, including nicotine, cotinine, benzopyrenes, and 4-(methylnitrosamino)-1-(3-pyridyl)-1-butanone [39,40,41].

A cohort study examining active and passive smoking in relation to HPV infection demonstrated that active smoking influences dysplastic lesions in the cervix uteri; however, it is not an independent risk factor for cervical lesions in individuals who do not smoke actively but are passively exposed to cigarette smoke [39,42].

Another population-based case–control study indicated that individuals exposed to passive cigarette smoke did not exhibit a significant risk relative to non-smokers; however, passive smokers with a history of consuming 20 pack-years of cigarettes encountered up to a sevenfold increased risk of cervical intraepithelial neoplasia (adjusted OR: 7.2 and 95% CI: 2.5–20.6) [43].

In a study involving African American women exposed to passive cigarette smoke for over one year and those who had never been exposed to cigarette smoke, a comparison was made in terms of high-grade squamous intraepithelial lesions (HGSILs) and low-grade squamous intraepithelial lesions (LGSILs). Those exposed to passive cigarette smoke were found to have a higher risk of high-grade lesions (OR: 1.5 and 95% CI: 1.0–2.2). Conversely, the same results were not observed in white American women (adjusted OR: 1.4 and 95% CI: 0.7–2.8) [44]. Consequently, it was noted that that study included a limited number of women, indicating the necessity for epidemiological studies including a larger group of individuals diagnosed with precancerous or invasive cancer. A study conducted in China examined the relationship between passive smoking exposure and high-grade cervical intraepithelial neoplasia lesions, revealing that the risk of developing HGSILs was 1.57 times higher in individuals exposed to passive cigarette smoke than in those not exposed to cigarette smoke (95% CI: 1.05–2.35). In women with significant exposure to both passive and active smoking, the risk of developing HGSILs was 4.67 times higher (95% CI: 1.17–18.70). In women with HPV positivity and exposure to passive cigarette smoke, the risk of developing HGSILs was 5.28 times higher (95% CI: 2.25–14.52) than in HPV-negative non-smokers. The study indicated that the risk of developing HGSILs was 4.04 times higher in individuals exposed to passive cigarette smoke during adolescence than in non-smokers (95% CI: 1.44–11.37). Based on the result of the study, the authors stated that exposure to passive cigarette smoke was an independent risk factor for the development of HGSILs. The authors also stated that in the middle age group in particular, the risk of developing advanced-stage cervical neoplastic lesions increased even more as the duration and dose of smoking exposure increased over 20 years and when HPV positivity was added [45].

Our study revealed that the rate of abnormal colposcopic biopsy results among individuals with over 20 pack-years of smoking was 2.19 times greater than that of non-smokers. Furthermore, those with more than 13 pack-years of smoking exhibited a 2.08 times higher rate of abnormal histopathology in the ECC results. Consistent with the literature, smoking was found to elevate the rates of cytological and histopathological abnormalities in the cervix. The limitations of our study include its retrospective nature, the absence of data regarding the age of first coitus and the number of sexual partners among female smokers, a lack of clear knowledge on HPV vaccination rates, and a lack of information on whether the women were affected by passive smoking. Additionally, the enrolment of women from three distinct centers and the performance of the pathological examinations at these sites may suggest a lack of homogeneity in our results. However, the assessments were performed by a clinician specialized in gynecological oncology and a pathologist experienced in gynecological oncology across the three major training and research clinics, thus mitigating this limitation. Furthermore, other limitations of our study include the inability to measure the quantity of nicotine in the body or cervix uteri of the smoking women, as well as the fact that all of the women presented to hospitals and thus do not represent the general population.

In addition, a limitation of this study is that all our cases were positive for high-risk HPV infection and were matched in terms of age, gravida, parity, BMI, additional diseases, and alcohol use rates. However, this study clearly revealed the threshold value of smoking in terms of pack-years and included a sufficient number of cases, which demonstrates the strengths of our study.

## 5. Conclusions

The primary etiological factor for pre-neoplastic and neoplastic changes in the cervix uteri is high-risk HPV infection. This infection is noted to persist and progress, leading to an escalation in abnormal cytological and histopathological changes in the cervix uteri, particularly in individuals with prolonged exposure to and a high pack-year history of cigarette smoking. We believe that it is critical to take the required steps to lower smoking rates in order to reduce the effects of HPV infection, which is being attempted to be controlled through routine screening programs across the world in order to improve women’s health.

## Figures and Tables

**Table 1:** Case–control matching parameters.

		Smoker				Total (N = 624)		
		Yes (n = 312)		No (n = 312)				*p*
Parity (n. %)	No	81	26%	81	26%	162	26%	1.000
	1–2	122	39%	122	39%	244	39%	
	>2	109	35%	109	35%	218	35%	
Gravida (n. %)	No	80	26%	80	26%	160	26%	1.000
	1–2	79	25%	79	25%	158	25%	
	>2	153	49%	153	49%	306	49%	
Alcohol (n. %)	Yes	118	38%	118	38%	236	38%	1.000
	No	194	62%	194	62%	388	62%	
Age (Mean ± SD)		47.1 ± 8.8		47.2 ± 8.5		47.1 ± 8.6		0.904

**Table 2:** Analysis of demographic, clinical, and cervical cytological data of the women.

	Smoker				Total (n = 624)				
	Yes (n = 312)		No (n = 312)				*p*	OR (95%CI)	*p * ^¥^
BMI, _Med (IQR)_	23.4	(20.7–30.1)	26	(22.1–32.1)	25	(21.2–31.1)	<0.001	–	–
CRP, _Med (IQR)_	5.2	(3.5–9.8)	4.2	(3.1–6.3)	4.3	(3.2–8.4)	<0.001	–	–
Fast. blood glucose	103	(89–121)	103	(86–122)	103	(88–121.5)	0.292	–	–
Education									
None	32	10%	38	12%	70	11%	<0.001	–	–
Primary School	144	46%	171	55%	315	50%			
High School	98	31%	94	30%	192	31%			
University	38	12%	9	3%	47	8%			
Menopause									
Yes	146	47%	140	45%	286	46%	0.630	1.08 (0.79–1.48)	0.630
No	166	53%	172	55%	338	54%		1	
Comorbidity									
Yes	139	45%	83	27%	222	36%	<0.001	2.22 (1.58–3.1)	<0.001
No	173	55%	229	73%	402	64%		1	
Abortion									
Yes	100	32%	89	29%	189	30%	0.338	1.18 (0.84–1.66)	0.338
No	212	68%	223	71%	435	70%		1	
Dilation and curettage									
Yes	66	21%	62	20%	128	21%	0.692	1.08 (0.73–1.6)	0.692
No	246	79%	250	80%	496	79%		1	
Contraception									
Yes	67	21%	87	28%	154	25%	0.063	0.71 (0.49–1.02)	0.064
No	245	79%	225	72%	470	75%		1	
Cervical cytology									
NILM	44	14%	91	29%	135	22%	<0.001	–	–
ASC-US	60	19%	68	22%	128	21%			
LGSIL	107	34%	85	27%	192	31%			
HGSIL	59	19%	45	14%	104	17%			
ASC-H	30	10%	12	4%	42	7%			
AGC-NOS	11	4%	9	3%	20	3%			
AIS	1	0%	2	1%	3	0%			

OR = odds ratio; Med = median; IQR = interquartile range; ^¥^ = *p*-value estimated using the Mantel–Haenszel common odds ratio. BMI: body mass index; CRP: C-reactive protein; NILM: negative intraepithelial neoplasia or malignancy; LGSIL: low-grade intraepithelial lesion; HGSIL: high-grade squamous intraepithelial lesion; ASC-H: atypical squamous cells—cannot exclude high-grade squamous intraepithelial lesion; AGC-NOS: atypical glandular cells not otherwise specified; and AIS: adenocarcinoma in situ.

**Table 3:** Evaluation of colposcopic and endocervical curettage histopathological results in smokers and non-smokers.

	Smoker				Total (n = 624)				
	Yes (n = 312)		No (n = 312)				*p*	OR (95%CI)	*p * ^¥^
Colposcopy									
HGSIL-AGC-NOS-CIS	87	28%	72	23%	159	25%	0.092	–	–
LGSIL	174	56%	169	54%	343	55%			
Chronic cervicitis	51	16%	71	23%	122	20%			
ECC-1									
HGSIL-AGC-FN-CIS	30	10%	33	11%	63	10%	<0.001	–	–
LGSIL	99	32%	55	18%	154	25%			
Chronic cervicitis—negative (no dysplasia)	183	59%	224	72%	407	65%			
ECC-1^x^									
HGSIL-AGC-FN-CIS-LGSIL	129	41%	88	28%	217	35%	0.001	1.79 (1.28–2.51)	0.001
Chronic cervicitis—negative (no dysplasia)	183	59%	224	72%	407	65%		1	
Leep conization									
HGSIL	74	85%	53	73%	127	79%	na	–	–
LGSIL	9	10%	18	25%	27	17%			
Chronic cervicitis	1	1%	1	1%	2	1%			
CIS-Cervical Ca	3	3%	1	1%	4	3%			
ECC-2									
HGSIL	33	38%	8	11%	41	26%	<0.001	–	–
LGSIL	31	36%	33	45%	64	40%			
Chronic cervicitis	17	20%	16	22%	33	21%			
Cervical Cancer	1	1%	0	0%	1	1%			
Negative (no dysplasia)	5	6%	16	22%	21	13%			

^¥^ = *p*-value estimated using the Mantel–Haenszel common odds ratio; na = not available. ECC-1^x^: comprising normal cellular changes and abnormal cellular changes in the two groups. HGSIL: high-grade squamous intraepithelial lesion; LGSIL: low-grade intraepithelial lesion; AGC-FN: atypical glandular cell favor neoplasia; CIS: carcinoma in situ; ECC: endocervical curettage; and LEEP: loop electrosurgical excision procedure.

**Table 4:** ROC analysis of colposcopy and endocervical curettage results according to smoking pack-years.

Variable	Classification	AUC	*p*_(Area=0.5)_	J_Youden index_	Cutoff Value	Sensitivity	Specificity
Smoking Pack-Years	ECC-1	0.586	0.008	0.14	>13	81%	32%
Smoking Pack-Years	Colposcopy	0.592	0.025	0.17	>20	40%	76%

ROC = receiver operating characteristic and AUC = area under the curve.

**Table 5:** Risk analysis of colposcopic biopsy results according to smoking pack-years.

	Colposcopy				Total				
	HGSIL-AGC/NOS-CIS-LGSIL		Chronic Cervicitis				*p*	OR (95%CI)	*p * ^¥^
Smoking pack-years									
>20	105	40%	12	24%	117	38%	0.036	2.19 (1.09–4.37)	0.027
≤20	156	60%	39	76%	195	63%		1	
Total	261	100%	51	100%	312	100%			

OR = odds ratio and ^¥^ = *p*-value estimated using the Mantel–Haenszel common odds ratio.

**Table 6:** Risk analysis of ECC results according to smoking pack-years.

	ECC-1				Total				
	HGSIL-AGC/FN-CIS-LGSIL		Chronic Cervicitis—Negative (No Dysplasia)				*p*	OR (95%CI)	*p * ^¥^
Smoking pack-years									
>13	105	81%	124	68%	229	73%	0.007	2.08 (1.21–3.58)	0.008
≤13	24	19%	59	32%	83	27%		1	
Total	129	100%	183	100%	312	100%			

OR = odds ratio and ^¥^ = *p*-value estimated using the Mantel–Haenszel common odds ratio.

## Data Availability

The datasets used and/or analyzed during the present study are available from the corresponding author upon reasonable request.
